# Bioactive Glasses: Frontiers and Challenges

**DOI:** 10.3389/fbioe.2015.00194

**Published:** 2015-11-30

**Authors:** Larry L. Hench, Julian R. Jones

**Affiliations:** ^1^Department of Biomedical Engineering, Florida Institute of Technology, Melbourne, FL, USA; ^2^Department of Materials, Imperial College London, London, UK

**Keywords:** Bioglass, bioactive glass, inorganic/organic hybrids, sol–gel, scaffold, regenerative medicine, tissue engineering, synthetic bone grafts

## Abstract

Bioactive glasses were discovered in 1969 and provided for the first time an alternative to nearly inert implant materials. Bioglass formed a rapid, strong, and stable bond with host tissues. This article examines the frontiers of research crossed to achieve clinical use of bioactive glasses and glass–ceramics. In the 1980s, it was discovered that bioactive glasses could be used in particulate form to stimulate osteogenesis, which thereby led to the concept of regeneration of tissues. Later, it was discovered that the dissolution ions from the glasses behaved like growth factors, providing signals to the cells. This article summarizes the frontiers of knowledge crossed during four eras of development of bioactive glasses that have led from concept of bioactivity to widespread clinical and commercial use, with emphasis on the first composition, 45S5 Bioglass^®^. The four eras are (a) discovery, (b) clinical application, (c) tissue regeneration, and (d) innovation. Questions still to be answered for the fourth era are included to stimulate innovation in the field and exploration of new frontiers that can be the basis for a general theory of bioactive stimulation of regeneration of tissues and application to numerous clinical needs.

## Introduction

It is an honor to present this opening paper in this special journal issue devoted to frontiers of inorganic biomaterials. Our contribution focuses on the frontiers and unmet challenges of bioactive glasses. It is now nearly 50 years since the discovery of bioactive glasses bonding to living bone (Beckham et al., 1971; Hench et al., 1971; Hench and Paschall, 1973; Wilson et al., 1981). Many advances have been made in understanding mechanisms of bonding of this special compositional range of glasses to both bone and soft connective tissues. Numerous published reviews and books have documented these advances (Hench, 1991, 1998, 2015; Hench and Polak, 2002; Hench et al., 2004; Rahaman et al., 2011; Jones, 2013). In the last decade, the primary clinical applications of bioactive glasses have involved turning on the body to repair its own bone, a process called *osteostimulation*, a term approved by the United States Food and Drug Administration (FDA). *Osteostimulation* refers to the activation of progenitor cells in the body, by a material or its dissolution products, producing more bone. The claim is based on *in vivo* data (Oonishi et al., 2000) that showed that Bioglass stimulates more rapid bone repair than other bioactive ceramics and the *in vitro* studies that revealed why this occurred, which was due to the dissolution products stimulating seven families of genes in primary human osteoblasts (Xynos et al., 2000a,b, 2001).

A recently published review summarizes the questions answered in four eras of development of bioactive glasses from the discovery in 1969 to the present, 2015 (Hench, 2015). The eras of development of bioactive glasses are

(A) Era of Discovery (1969–1979);
(B) Era of Clinical Application (1980–1995, C);
(C) Era of Tissue Regeneration (1995–2005);
(D) Era of Innovation (2005–2025).

Several important unanswered questions for the fourth era were suggested in the review (Hench, 2015). Each of these unanswered questions is at the frontier of understanding and controlling the interaction of bioactive glasses in the living body. The objective of this introductory paper is to discuss these questions further, suggest potential research directions that can answer them to move the frontiers of the field forward to achieve even more clinical applications for an aging population.

## What Are Frontiers?

**Figure 1:**
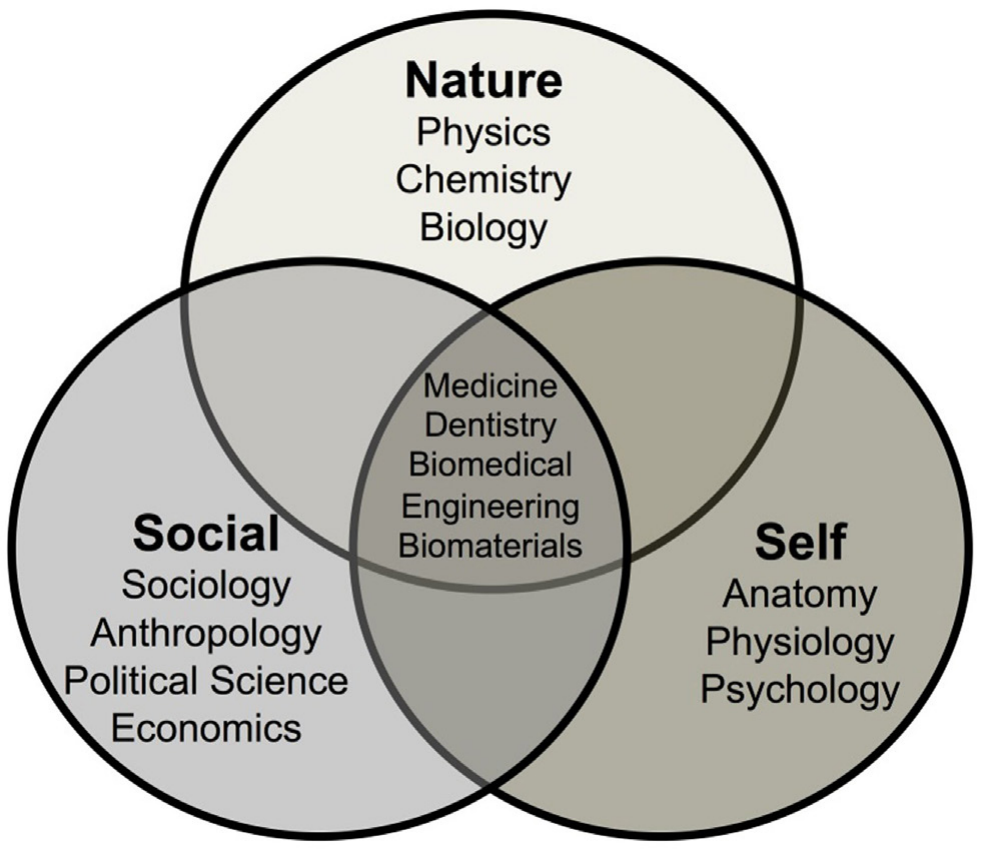
**Three realms of human knowledge**.

First, it is important to discuss the concept of frontiers of knowledge in general and the frontiers of biomedical materials specifically. We can divide human knowledge into three overlapping and intersecting realms of knowledge: *Nature*, *Self*, and *Social* (Figure 1).

The first field of knowledge, called *Nature*, evolved over millennia as humans strived to understand the natural forces that influenced their lives. The subject was first titled natural history. At one time, it was suggested that natural historians such as Sir Francis Bacon possessed within his own mind most of what was known about the natural world at that time. Now in the twenty-first century, it is impossible for any one individual to know or understand even a very small fraction of the knowledge of nature. The field has been divided into the major scientific disciplines of physics, chemistry, and biology then subsequently subdivided into an ever-increasing number of subdisciplines, such as astronomy, astrophysics, cosmology, quantum mechanics, solid-state physics, inorganic chemistry, organic chemistry, biochemistry, molecular biology, etc. Although enormous depth of understanding of these topics has been achieved, there are still many frontiers in the knowledge of nature. These frontiers are at the boundary between certainty and uncertainty. Those boundaries exist at the extremes of scale of distance and time limits of our universe. Distances of very small, sub nanometer size, and very large, light years in dimension, comprise the bounds of uncertainties. There are discoveries every year that push back the age of the universe and the complexity and beauty of the subatomic particles that were created during the “Big Bang” beginning of the universe that comprise the atomic and molecular-based world that we live in today.

The knowledge of *Self* also emerged during the last few millennia as a set of disciplines, such as anatomy, physiology, and psychiatry. Intersections between the knowledge of *Self* and *Nature* have become ever more blurred in today’s scientific community with the application of many of the techniques used to explore the natural universe also applied to understanding the human body, the brain, and the mind. The frontiers of knowledge of *Self* are still largely unexplored and the origins of thought, memory, and emotions are active subfields of investigation. Advances in the understanding of *Nature* and *Self* have made it possible to control the life and the death of billions of humans.

The third sphere of knowledge, evolved over the last few hundred years, can be considered *Social* knowledge. Subdisciplines, such as sociology, anthropology, economics, and political science, have been developed to attempt to explain the complex interrelationships between individuals. Social knowledge includes small group interactions, such as couples, to large-scale interdependence of communities involving millions of individuals. Levels of uncertainty in the field of social knowledge are extremely high. This is because of the difficulty of predicting the behavior of large number of individuals interacting together. To become a science, it is necessary to achieve repeatable observation, verification, and quantification, followed by predictability. Such criteria are met in the natural sciences and the ever-increasing knowledge of *Self*.

However, there are high levels of unpredictability in the area of social knowledge. Thus, world conflicts continue to occur with enormous toll on human suffering and life without a means to predict or prevent such calamities. Unpredictable political changes, such as the breakup of the Soviet Republic were seldom, if at all, predicted by social scientists. Even breakups of interpersonal relationships of couples are, for the most part, unpredictable. Likewise, it is very difficult to predict the impact of a new medical therapy on the behavior of a large population. Self-delusion and susceptibility to persuasion can easily warp the attitude of large numbers of individuals and replace logical reasoning in decision making. As an example, many surgeons find it difficult to accept that a bioactive synthetic bone graft can be equal or superior to autogeneous bone (bone transplanted from another part of the patient), even though clinical studies have shown that to be the case for some applications, even though the autograft leads to donor site morbidity.

Of particular concern in this introductory paper is a discussion of the area where the three realms of knowledge overlap and intersect, as illustrated in Figure 1. Medicine, Dentistry, Biomedical Engineering, and Biomaterials lie at the intersection of the three realms of knowledge. This region could also be broadly named Healthcare. Here, the uncertainties of each realm of *Nature*, *Self*, and *Social*, are additive and perhaps even multiplicative. Thus, it becomes nearly impossible to predict the effects on long-term survivability (20–40 years) of a change in a biomaterial or device in a single individual. This is because the healthcare predictions, derived from overlapping regions of the three realms of knowledge, are based upon statistical results of the survivability of a large number of patients. The uniqueness of an individual is not reflected in statistical data, only within the distribution of results.

This fact is extremely important to recognize, as the field of repair and regeneration of the human body increases to deal with an aging population numbering in the hundreds of millions. It is important for the entire healthcare community, and the general public, to recognize that there are no such things as miracle materials or miracle cures. There is always the possibility of failure. Failure is not necessarily the fault of any individual, surgeon, company, or hospital. Failures of materials, devices, and biotechnology are a natural consequence of the large-scale complexity of the human body and its intricate interactions in a social environment where outside influences affect uncertainty of the quality of life of the individual as well as the length of life.

Let us discuss one example of an unmet challenge to illustrate the impact of the uncertainties of these overlapping regions of knowledge on inorganic biomaterials device development. During the last 40 years, numerous research efforts have been made to develop a long-term stable (not biodegradable) load-bearing replacements for diseased, damaged, or missing bone. The closest bioceramic to achieve this objective was the apatite–wollastonite (A/W) bioactive glass–ceramic, Cerabone, developed in Japan, at the University at Kyoto, by professors Yamamuro, Kokubo, Nakamura, and colleagues (Kokubo et al., 1990). Tens of thousands of successful Cerabone implants were made and implanted for a variety of orthopedic applications in Japan, especially in spinal repair. Excellent clinical success was achieved for all of the devices. However, a very high stiffness (elastic modulus) led to concern about long-term stress shielding in high load-bearing applications. Stress shielding occurs when load is transmitted through the implant, and it is not transmitted to the surrounding bone. When bone is not loaded, it loses volume as the body removes it through osteoclast cell activity. A high production cost also limited commercial interest. The product was not introduced internationally and is no longer on the market. Thus, the goal of replacing load-bearing cortical bone is still an unmet challenge.

Our issues of concern are the uncertainties associated with the intersections of the three worlds of knowledge. The laws of nature make it possible to perform accurate mechanical testing of a new biomaterial, such as a potential load-bearing bioactive ceramic. Mechanical testing can be extended to a sufficiently large number of test devices to establish the distribution of results and strain rate dependencies of strength can lead to lifetime prediction diagrams of the mechanical behavior under particular levels of load. The science behind the knowledge of *Self* now makes it possible to obtain quantitative computed tomography (CT) data (Midha et al., 2013), and by use of rapid prototyping replicate precisely, the anatomical shape needed for a device made of a new load-bearing bioactive ceramic (Brie et al., 2013). The science of ceramic, glass, and glass–ceramic processing is sufficiently advanced to make individual components by rapid prototyping or computer-guided machining at reasonable cost. Developments, such as 3-D printing, make it possible to manufacture anisotropic microstructures that mimic the structure of cortical bone as well as trabecular bone (Fu et al., 2011a,b).

Uncertainties, however, have great impact on the economics of the overlap between *Nature*, *Self*, and *Society*. Limitations on new medical product development come in several forms. Achieving governmental regulatory approval of a new device that must last for many years requires a highly rigorous set of simulation testing and large monetary investment. The keyword here is simulation. Simulated body solutions are a standard use in the bioceramics testing field and have been adopted as international and regulatory standards (Macon et al., 2015). However, the non-cellular simulated body fluids do not lead to an ability to predict in long term the effect of a physiological body environment on a material or device that is exposed to a complex mixture of mechanical loads (Bohner and Lemaitre, 2009). It is well known that bone cells respond to mechanical cues and the architecture and the quality of bone that forms is dependent on those cues. Multiaxial fatigue data can be generated under simulated physiological conditions, but such environments do not embrace the uncertainty of the effect of the living bone – bioactive ceramic contact area and its changes with time and physiology of the patient.

Especially important is the fact that there is no way to predict the effect of age and load distribution on the mechanical properties of the loaded bone bonded to the bioactive ceramic. Consequently, in order to have a sufficiently acceptable set of preclinical data, it is necessary to establish reasonably equivalent animal data for the regulatory authorities. Approval for clinical application of an innovative bioactive load-bearing bioceramic will require large animal data. This is where the overlap of *Nature*, *Self*, and *Social* is especially important because the cost of producing large animal data for a statistically significant number of implants is very high.

The costs escalate after successful animal data has been generated because most regulatory agencies will require clinical trials. The number is large because there is no predicate load-bearing cortical bone implant to establish equivalence under the FDA 510K provisions. It is very difficult to predict the cost of clinical trials because the survivability for approval must surely be established for a minimum of three, and more than likely 5 years. Thus, the cumulative cost of bringing a new product, such as a new bioactive ceramic material, into the market is in the millions of dollars. Although there are tens of thousands such devices potentially needed annually, it is very hard to calculate the potential cost/profit or risk/reward ratios. These limitations and barriers to achieving frontiers of clinical use are independent of the successful development of the biomaterial that satisfies the ideal combination of properties needed for cortical bone; i.e., strength, toughness, fatigue resistance, bioactivity, and elastic modulus that do not shield the bone from stress following bonding to bone.

The above example illustrates our opinion that emphasis on “improved” bioactive ceramics, where the primary function of the material is to replace the diseased, damaged, or missing tissue is unlikely to have many successes that are economically viable. These considerations lead to the conclusion that the most significant long-term frontier is expansion of efforts in the field toward the frontiers of regenerative medicine. This era of innovation will be discussed briefly next. The critical unmet challenges and frontiers will be discussed later. However, let us first look at what can be considered a frontier in this field of inorganic biomaterials.

The word *frontier* implies exploring the unknown hoping to find within the unknown something new and useful. In directed research, such as biomaterials, new and important developments are driven by clinical need and limited by economics and long-term survivability. Deciding which frontiers to explore is a difficult and demanding step for a research group or company. The history of the field has shown that there are indeed very few frontiers that have been crossed, although there have been thousands of efforts to achieve long-term improvement of biomaterials in general.

An example from the field of bioactive glasses and glass–ceramics can be useful in establishing what is and what is not a frontier of research in the field. The very first material that was found to form a bond with bone was the original bioactive glass composition, 45S5 Bioglass (45 wt% SiO_2_, 24.5 wt% CaO, 24.5 wt% Na_2_O, and 6 wt% P_2_O_5_) (Hench et al., 1971). Much of the time in the era of discovery was devoted to understanding the mechanisms of bonding and the nature of the bonds between the glass and bone and soft tissue (Hench and Polak, 2002). This can be considered a major frontier because up until the time of this discovery, it was assumed that all foreign materials would be isolated from the living tissue by a thin acellular fibrous capsule. The discovery showed that encapsulation was not a fundamental restriction of the response of the body to foreign material. When rapid reactions occur at the surface of a bioactive glass or glass–ceramic, the biologically active hydroxyapatite (HA) layer quickly masks the material from immune cells. The cellular recognition mechanisms respond to it as if it were a layer of newly mineralizing bone: the cells attach and extracellular matrix (ECM) is produced, mineralization proceeds to completion and newly formed bone is strongly anchored to the surface of the material with an interfacial bond strength equal to or greater than the natural bone (Hench et al., 1971). Another frontier was discovery of the bonding of the most bioactive of the Bioglasses to soft connective tissues as well as bone through an equivalent mechanism of surface reactions to form a hydroxyl-carbonate apatite layer but with a thicker bonding interface (Wilson and Noletti, 1990).

The compositional boundary between bonding to bone and non-bonding was found to be in the range of 60 wt% silica (Hench, 1998). Effects of additional oxide compositions on the details of the compositional boundaries have been looked at extensively in the decades since (Hoppe et al., 2011). Some investigators proclaim that addition of other oxides to the bioactive glass to enhance the bone bonding is searching the new frontier. This objective is open to question because the measure of frontier advances is delivery of clinical products. Small incremental advances showing a few percent more bone growth in a 30-day period of time is questionable as frontier research, as the small increase will not warrant the investment required to get the new material to market. However, the realization that the dissolution ions caused osteostimulation was the crossing of an important frontier: cell stimulation by a synthetic material without organic growth factors. If additional therapeutic benefits of other cations can be proven, there is great potential to use bioactive glass as a reservoir for sustained delivery of active ions that can be specific to different medical conditions. An example is strontium oxide, where controlled release of strontium ions, from the glass, is thought to be beneficial for osteoporosis as it can slow osteoclast activity (Lao et al., 2008; Gentleman et al., 2010; Autefage et al., 2015).

What is frontier research in bioactive materials? Returning back to the discussion of long-term survivability of load-bearing long bone implants, research done up to now has not delivered such a material. Consequently, the concept of tissue regeneration to enhance bone formation that is capable of long-term load-bearing is now at the highest level of frontier investigations. This is because the concept of tissue regeneration is to use the material not to replace the diseased, damaged, or missing part of the body, but instead activates the body’s own repair mechanism so that the tissue that is grown is replicating both biochemically and biomechanically the original load-bearing tissue. This eliminates the problems of stress shielding and particularly the problem of remodeling of the material or interface when the load distribution changes or health deteriorates. Thus, an active frontier area includes developing an ideal bioactive scaffold for bone that is capable of providing short-term strength with high reliability that is transformed into load-bearing bone and then resorbed. This is one of the most significant levels of frontiers and the progress to achieve it is a highlight of the decade of innovation.

Designing hybrid biomaterials that are bioactive and have controlled rates of resorption and can be molecularly tuned to produce particular combinations of mechanical properties is a major goal of the era of innovation, as discussed in a recent review (Jones, 2013). Success in achieving an ideal scaffold for bone as a frontier would have a large impact on the field. It would open exploration to achieve an ideal scaffold for cartilage regeneration that could provide a long-term solution to the extensive revisions now required for replacement of total hip and knee prostheses due to the biomechanical limitations discussed above. The frontier of designing bioactivity to activate the genetic repair mechanisms for specific lineages of connective tissues is at the highest level of frontier research and will be emphasized in the list of unmet challenges to follow. Of course, to cross the frontiers, the new devices must be translated to clinical products.

## ERA of Discovery (1969–1979) Frontiers

The most significant frontier was the discovery in 1969 by Hench, Splinter, Allen, and Greenlee that certain compositions of Na_2_O–CaO–P_2_O_5_–SiO_2_ glasses formed a strong, adherent bond to bone (Hench et al., 1971). These biomaterials have become known as “bioactive,” reacting in the physiological environment to form a bond between an artificial material and living tissue. Studies showed stable and strong bonding between bone and soft tissues in a wide range of mammals: mice, rats, guinea pigs, rabbits, dogs, sheep, pigs, monkeys, and baboons. A stable bone-bonded implant in the anterior region of the mandible of a baboon after 4 years of functional use was reported, one of the longest *in vivo* studies of biomaterials in primates ever published (Stanley et al., 1981).

The second frontier was development of *in vitro* and *in vivo* tests that established the mechanisms and limits of bonding of bioactive glasses and glass–ceramics to bone. The *in vitro* tests showed that the 45S5 Bioglass composition (see Table 1) developed a HA layer in test solutions. This HA phase developed on the surface of the implants *in vitro* was equivalent to the interfacial HA crystals observed *in vivo* by Dr. Greenlee’s transmission electron micrographs of the bonded interface. The HA crystals *in vivo* were bonded to layers of collagen fibrils produced at the interface by osteoblasts. The chemical bonding of the HA layer to collagen created the strongly bonded interface (Beckham et al., 1971; Hench et al., 1971; Hench and Paschall, 1973).

**Table 1:** **Composition and properties of bioactive glasses and glass–ceramics used clinically for medical and dental applications**.

Composition (wt%)	45S5 Bioglass (NovaBone, Perioglas, NovaMin, Biogran)	S53P4 (AbminDent1, BonAlive)	A–W glass–ceramic (Cerabone)
Na_2_O	24.5	23	0
CaO	24.5	20	44.7
CaF_2_	0	0	0.5
MgO	0	0	4.6
P_2_O_5_	6	4	16.2
SiO_2_	45	53	34

During the era of discovery, a series of questions was addressed (Hench, 2015). Some of the key questions are summarized here. Question: What is the nature of the bioactive bond? Answer: hydroxycarbonate apatite (HCA) crystals bonded to collagen fibers (Beckham et al., 1971; Hench et al., 1971; Hench and Paschall, 1973). Question: What mechanisms are involved in HCA formation? Answer: five surface reactions at the glass surface occur (cation exchange, Si–OH group formation, on which amorphous calcium phosphate phase deposits, crystallizing to HCA, which binds to collagen). Question: How strong is the bond? Answer: stronger than the host bone (Piotrowski et al., 1975). Question: What compositions of glass can form the bond? 45S5 Bioglass is composed of SiO_2_–CaO–Na_2_O–P_2_O_5_ (Table 1). In this system, bonding to both bone and soft tissue is possible at 52 wt% SiO_2_ (Wilson et al., 1981) and between 52 and 60% SiO_2_ bonding is only to bone (Hench, 1998).

Two important aspects of the frontiers were explored in the Era of Discovery. First, the methodology for investigating the reactive glass surface and bonded interfaces of bioactive implants with living tissues had to be developed. There was no precedent for such analyses. Examples are instrumental techniques such as infrared reflection spectroscopy, developed by Sanders and Hench (1973), and applied to bioactive glasses and cryogenic Auger electron spectroscopy (AES), developed by Ohuchi, Pantano, Ogino, and Hench (Clark et al., 1976; Ogino and Hench, 1980; Ogino et al., 1980). At this stage, tests were conducted primarily on bulk samples or as bioactive coatings on metal, e.g., Co–Cr alloys, or ceramic (e.g., alumina) implants. It was assumed that the eventual applications of bioactive bonding would be to replace a diseased or damaged bone. The second Era of Clinical Applications was based upon this knowledge.

## ERA of Clinical Application Frontiers (1980–1995)

An important frontier to cross was clinical translation. The discovery by Wilson et al. (Wilson et al., 1981; Wilson and Noletti, 1990) that Bioglass could bond to soft tissue paved the way for development of the first bioactive glass clinical applications that required both stable bone and soft tissue interfaces: the MEP (middle ear replacement prostheses) (Merwin et al., 1982) and ERMI, endosseous ridge maintenance implants (Stanley et al., 1997). These devices had the objective of replacing diseased, damaged, or missing body parts that require stable bonding to both soft tissues and bone. At the time, most other types of middle ear prostheses were lost by extrusion after a few years. In contrast, Bioglass middle ear devices formed a stable bond to both bone, such as the stapes footplate and the soft tissues of the tympanic membrane and thus remained stable for more than 10 years as reported in follow-up studies at both the University of Florida and Guy’s Hospital in London (Rust et al., 1996). Equivalent long term, >10 years, success of the Bioglass ERMIs were reported by Stanley et al. Alternative Class B bioactive implants made of synthetic HA were lost by extrusion or exfoliation from the jaw after only a few years post implantation. In contrast, 45S5 Bioglass implants maintained stable bonding in alveolar bone and a stable gingival interface for long term and maintained thickness of the bone without resorption generally experienced by denture wearers (Wilson et al., 1993; Stanley et al., 1997).

A second frontier crossed by Wilson et al. (1981) was the collation of results of sixteen *in vitro* and *in vivo* tests that established the safety of use of particulate forms of Bioglass in addition to the bulk implants (Wilson et al., 1981). These data provided the basis for ethical committee’s approval of the use of Bioglass in clinical trials at the University of Florida and Guy’s Hospital in London, as well as application for regulatory approval of commercial sales of these devices by the FDA and a CE mark from the EU. This led to the use of Bioglass in bone regeneration (Hench et al., 2004).

## ERA of Tissue Regeneration Frontiers (1985–2005)

**Figure 2:**
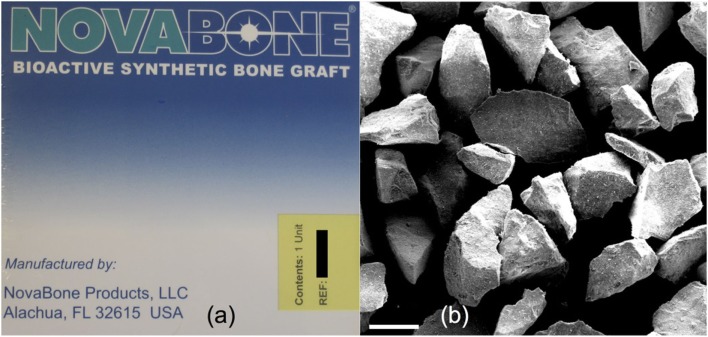
**(A)** Packaging of NovaBone (45S5 Bioglass) powder for orthopedic applications and **(B)** scanning electron micrograph of NovaBone particles. Modified with permission from Jones (2013).

The discovery of osteoproduction (osteostimulation) and the concept of using Bioglass particulate for regeneration of bone was the key frontier crossed that led to the Era of Tissue Regeneration. Wilson et al. described the effect of various sizes of Bioglass particulate on regeneration of bone in periodontal defects created in a monkey model (Wilson and Low, 1992). The seminal finding was the stimulation of new bone throughout the defect. Bone growth was initiated at the surface of the bioactive glass particles and rapidly formed connections between the particles regenerating a trabecular bone network that mimicked the original trabecular bone of the jaw prior to creating the defect. The study showed that there was an optimal rate of bone repair when a range of particle sizes of Bioglass was used. The results also showed that bone regeneration was sufficiently rapid that it prevented encapsulation of the site by epithelial tissues. The data provided the foundation for a clinical trial in patients at the University of Florida that led to FDA regulatory approval of the use of bioactive glass particulate for periodontal repair (Perioglas, NovaBone Products LLC, Alachua, FL, USA; Figure 2).

NovaBone was compared to autograft in posterior spinal fusion operations for treatment of adolescent idiopathic scoliosis (curvature of the spine) in 88 patients (Ilharreborde et al., 2008). NovaBone performed as well as autograft over 4 years but with fewer infections (2 versus 5%) and fewer mechanical failures (2 versus 7.5%).

The first non-45S5 composition to reach the market was S53P4 (Table 1), now known as BonAlive^®^ (BonAlive Biomaterials, Turku, Finland), which received European approval for orthopedic use as bone graft substitute in 2006. It has higher silica content; so bioactivity is expected to be lower than 45S5. Several clinical trials have been published from the work in Finland, importantly using the gold standard autograft as comparison, which is needed to convince surgeons. A mixture of granules with autologous bone allowed the implantation of titanium implants in previously damaged jaw bone and showed more rapid bone repair compared to autograft alone (Turunen et al., 2004). Clinical trials for severe spondylolisthesis (displacement of vertebrae) used granules of 1–2 mm. After 11 years, the fusion rate for the glass was 88% compared to 100% for autograft (Frantzen et al., 2011). Similar results were seen for treatment of osteomyelitis, where bone quality of the vertebrae is reduced due to bacterial infection (Lindfors et al., 2010a). BonAlive was also compared to autograft in the same patients in spondylodesis procedures for treatment of spine burst fractures. At 10-year follow-up, five out of 10 implants had full fusion compared to all 10 autografts (Rantakokko et al., 2012).

In tibial fractures, in which surgery was required to restore joint alignment, BonAlive particles (0.83–3.15 mm) were placed inside the subchondral bone defects with metallic fixation (Pernaa et al., 2011). Full weight bearing was allowed when radiographs indicated healing had occurred, so the implants were loaded, and 11-year follow-up showed similar bone regeneration compared to autograft. Some particles were still present at 11 years post operation (Heikkila et al., 2011). The lack of resorption of S53P4 may be due to glass composition, which has higher silica content than 45S5. Improvement over autograft was seen when BonAlive granules (1–4 mm) were used in post-tumor removal bone defects, with cortical bone thickness twice as thick as it was when autograft was used after 14 years (Lindfors et al., 2010b). Remodeling of the bone is slower than it was for autograft (e.g., after 12 months) (Lindfors et al., 2008).

A frontier that provided a scientific foundation for use of bioactive glass in bone regeneration was introduction of an appropriate *in vivo* model that allowed the quantification and comparison of bone regeneration for different bioactive materials. Use of the same model, and the fact that the model is appropriate, will accelerate translation of new medical devices. Quantification and comparison of the effect of bioactive glass on regeneration of bone was based upon a series of important studies conducted by Oonishi et al. in Osaka, Japan (Oonishi et al., 1997, 1999, 2000). The Oonishi investigations used a critical size defect in a rabbit femoral condyle model to compare rates of bone formation in the presence of different types of bioceramic particles of the same particle size. The studies showed there are more bones formed in just 1 week in the presence of 45S5 bioactive glass particulate than are formed when synthetic HA or other calcium phosphate ceramic particulates are placed in the same type of defect for several weeks. After several weeks of bone regeneration, there was almost twice as much new bones present in the defect-containing bioactive glass. By 12 weeks, the amount of bone regenerated by Bioglass particles matched that originally present in the site. Wheeler et al. demonstrated that the mechanical properties of the defect site were restored (Wheeler et al., 2000). Bioactive glasses and ceramics can, therefore, stimulate different rates of bone regeneration inside a bone defect, depending on the type of material (glass, ceramic, or glass–ceramic) and the composition and morphology of the device.

The next frontier was identifying what was really stimulating bone regeneration. While the *in vivo* data showed differences between implants, they were not answering the question why there were differences. Initially, the dissolution of the 45S5 Bioglass particles were thought to cause more bone formation by the HCA layer forming more rapidly and by the glass degrading, making more space for bone ingrowth. This was not the complete story though. Dissolution is important, but mainly because the dissolution ions act as signals to the cells. This was revealed through *in vitro* experiments that showed critical concentrations of Si and Ca ions released from the glasses stimulated cells at the genetic level. Seven families of genes were upregulated when primary human osteoblasts are exposed to the ionic dissolution products of bioactive glasses (Xynos et al., 2000a,b, 2001). The gene expression occurs within 48 h, and includes enhanced expression of more than twofold of seven families of genes. The dissolution products can direct the cycle of a mixed population of cells. Cells that are not capable of differentiation into a mature osteoblast phenotype are switched into apoptosis by the ionic stimuli, eliminating them from the culture environment within the first days of exposure to the bioactive stimuli. Upregulated genes encode nuclear transcription factors and cell cycle regulators (Xynos et al., 2001). Potent growth factors, especially insulin-like growth factor II (IGF-II), were increased by 3.2-fold along with IGF binding proteins and proteases that cleave IGF-II from their binding proteins.

Similar bioactive induction of the transcription of at least five ECM components (2- to 3.7-fold) and their secretion and self-organization into a mineralized matrix are responsible for the rapid formation and growth of bone nodules and differentiation of the mature osteocyte phenotype. Shifts in osteoblast cell cycles were observed as early as 6 h for most experiments, with elimination (by apoptosis) of cells incapable of differentiation. The remaining cells exhibited enhanced synthesis and mitosis. The cells quickly committed to generation of ECM proteins and mineralization of the matrix (Xynos et al., 2000a,b, 2001).

Similar results were seen for fetal osteoblasts, where critical concentrations of Bioglass dissolution products stimulated differentiation into mature phenotypes (Tsigkou et al., 2009). The roles of individual ions are partly understood: extracellular calcium ions increase IGF-II upregulation (Maeno et al., 2005; Marie, 2010) and glutamate production by osteoblasts (Valerio et al., 2009). Silica is released from Bioglass as silicic acid [Si(OH)_4_], which has been shown to stimulate collagen I production by osteoblasts (Reffitt et al., 2003). More detail on cellular response to individual ions is given in Hoppe et al. (2011).

## ERA of Innovation (2005–2025) Frontiers and Unmet Challenges

There are many challenges still ahead for the clinical use of bioactive glasses that require advances in a fourth era, an era of innovation. Significant scientific and technological issues remain unanswered.

### Frontier: Guidance of Stem Cells by Materials

Tissue regeneration through gene activation by controlled release of inorganic ions is a clinical reality that leads to enhanced osteogenesis. However, the role of the dissolution products on bone marrow-derived adult stem cells [mesenchymal stem cells (MSCs)] is more controversial, sometimes inducting osteogenic differentiation into osteoblast-like cells (Karpov et al., 2008; Brauer et al., 2010) and other times not (Reilly et al., 2007). Human adipose stem cells differentiated into osteogenic cells when cultured with bioactive glasses in the presence of osteogenic supplements (Haimi et al., 2009; Ojansivu et al., 2015). However, neither adipose nor bone marrow MSCs differentiated in the presence of submicron bioactive glass spheres (Labbaf et al., 2011; Tsigkou et al., 2014). The exact type and status of cell cycle of MSCs may be the reason for these differences.

An unmet challenge is to understand the fundamental mechanisms involved in ionic stimulation at the nucleus in the cell, of the many different cell types, that leads to upregulation or activation of genes. Another issue is that not all the articles explain exactly how the bioactive glass particles/dissolution products were applied or what supplements were used in the media. The fundamental mechanisms of stimulation of stem cell differentiation toward specific phenotypes must be understood to avoid potential tumorogenesis. A consolidation of data is needed for the frontier of stem cell guidance by bioactive glasses is still to be crossed.

### Unmet Need: Bioactive Glass Scaffolds as Clinical Products

**Figure 3:**
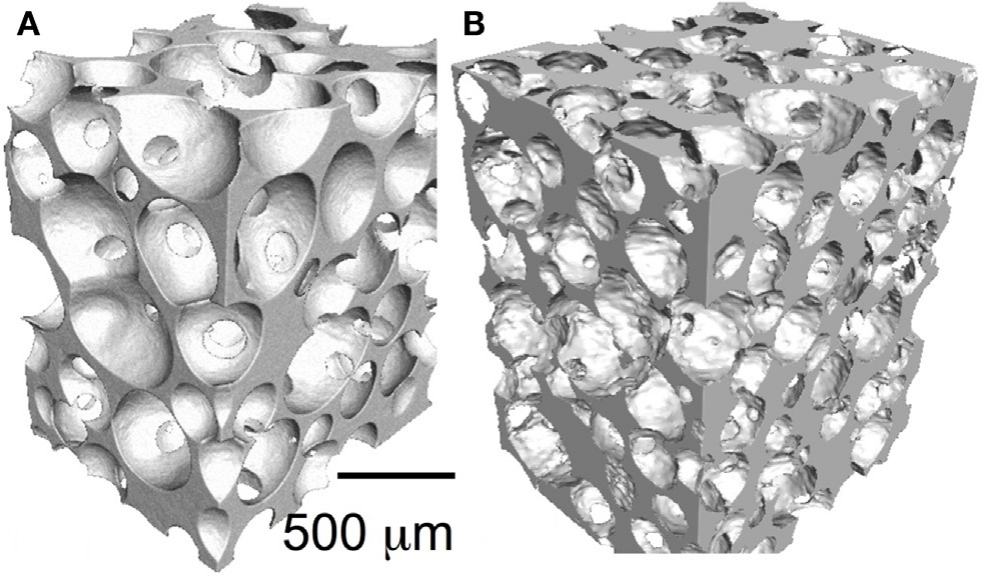
**X-ray microtomography images of bioactive glass scaffolds (A) sol–gel foam and (B) melt-derived gel-cast foam**. Modified with permission from Jones (2013).

Particles and putties containing a variety of bioactive glass particulates are in widespread clinical use, but surgeons sometimes require large interconnected macroporous scaffolds for regeneration of large bone defects. The porous architecture can guide bone regeneration, acting as temporary templates for tissue growth while allowing space for vascularization. At present, there are no large-scale porous bioactive glasses on the market. The reason is that it took until 2002 for the first porous bioactive glass scaffold with suitable pores to be developed (Sepulveda et al., 2002). This is because the original Bioglass 45S5 crystallizes as the particles are sintered together (Chen et al., 2006). Initially, this was overcome by avoiding sintering through the bottom-up sol–gel process, where gelation of nanoparticles in a sol (polycondensation) forms a glass network (Li et al., 1991). The room temperature gelation process allowed the introduction of a foaming step, with the aim of a surfactant, to produce interconnected pores with compression strength equivalent to porous bone (Jones et al., 2006). An X-ray microtomography image of a bioactive glass sol–gel foam scaffold is shown in Figure 3A.

More recently, melt-quenched glass scaffolds were produced through control of the sintering processing window by tailoring of the glass composition, which was achieved while maintaining bioactivity with new compositions, such as 13–93 (53 wt% SiO_2_, 6 wt% Na_2_O, 12 wt% K_2_O, 5 wt% MgO, 20 wt% CaO, and 4 wt% P_2_O_5_) (Brink, 1997; Fu et al., 2010) and ICIE16 (49.46 mol% SiO_2_, 1.07 mol% P_2_O_5_, 36.27 mol% CaO, 6.60 mol% Na_2_O, and 6.60 mol% K_2_O) (Elgayar et al., 2005; Wu et al., 2011). An ICIE16 scaffold is shown in Figure 3B. Now sol–gel and melt-derived scaffolds exist but none are being put forward for use by medical device companies, even though comparative *in vivo* studies show benefit over current commercial porous bioactive ceramics (Minaberry and Jobbagy, 2011). This is because the improvements in performance do not warrant the significant investment required to obtain FDA approval and upscale the manufacturing routes to commercial scale, as discussed above.

### Frontier: Tissue Engineered Constructs for Clinical Bone Regeneration

Tissue engineered constructs for replacement of large bone defects have been investigated for many years but are still not available as routine clinical products. Is it possible to achieve a stable vasculature *in situ* in tissue engineering constructs that can be maintained in culture before implantation or be generated *in vivo* following implantation? Tsigkou et al. demonstrated that it is possible in mice models (Tsigkou et al., 2010), but can it be translated to the clinic? Does the scaffold affect *in vitro* vascularization? Is the vascularization affected by mechanical load and changes of load with time? Numerous studies demonstrate bioactive stimulation of angiogenesis *in vitro*; however, many studies are on one cell type, often fibroblasts. Most of the studies look for expression of VEGF from the cells, e.g., from fibroblasts (Day et al., 2004; Day, 2005), which was dependent on the dose of Bioglass dissolution ions (Keshaw et al., 2005). Mitogenic stimulation of endothelial cells also occurred when they were cultured in the presence of Bioglass dissolution ions (Leach et al., 2006). Collagen/Bioglass 45S5 composites in rat calvaria also stimulated more neovascularization in 2 weeks than collagen alone (Leu et al., 2009), although similar results are not always seen in other studies. Extracellular calcium ions could be responsible for this effect (Aguirre et al., 2010). *In vitro* enhancement of angiogenesis has also been achieved by incorporating active ions, such as cobalt ions, in the glass network, which can trick the body into recognizing the implant site as hypoxic (low oxygen pressure). This triggers a cascade of processes to produce new blood vessels (Peters et al., 2005; Semenza, 2007; Azevedo et al., 2010, 2015). A question that needs answering is how long should there be presence of cobalt and a simulation of hypoxia in a bone defect for ideal bone regeneration? Medical device companies will also have to consider whether the benefits of cobalt ion release is worth the investment to claim the “drug-like” effects.

### Frontier: Regenerative Scaffolds That Are Truly Load Bearing

Load-bearing devices that can be used in orthopedics over the long term that can also regenerate living bone are still not available clinically. This would be a frontier crossed that would certainly warrant the investment of medical device companies. Is it feasible to produce and test bioactive implants that have predictable 20-year lifetime survivability under simulated load-bearing physiological conditions?

**Figure 4:**
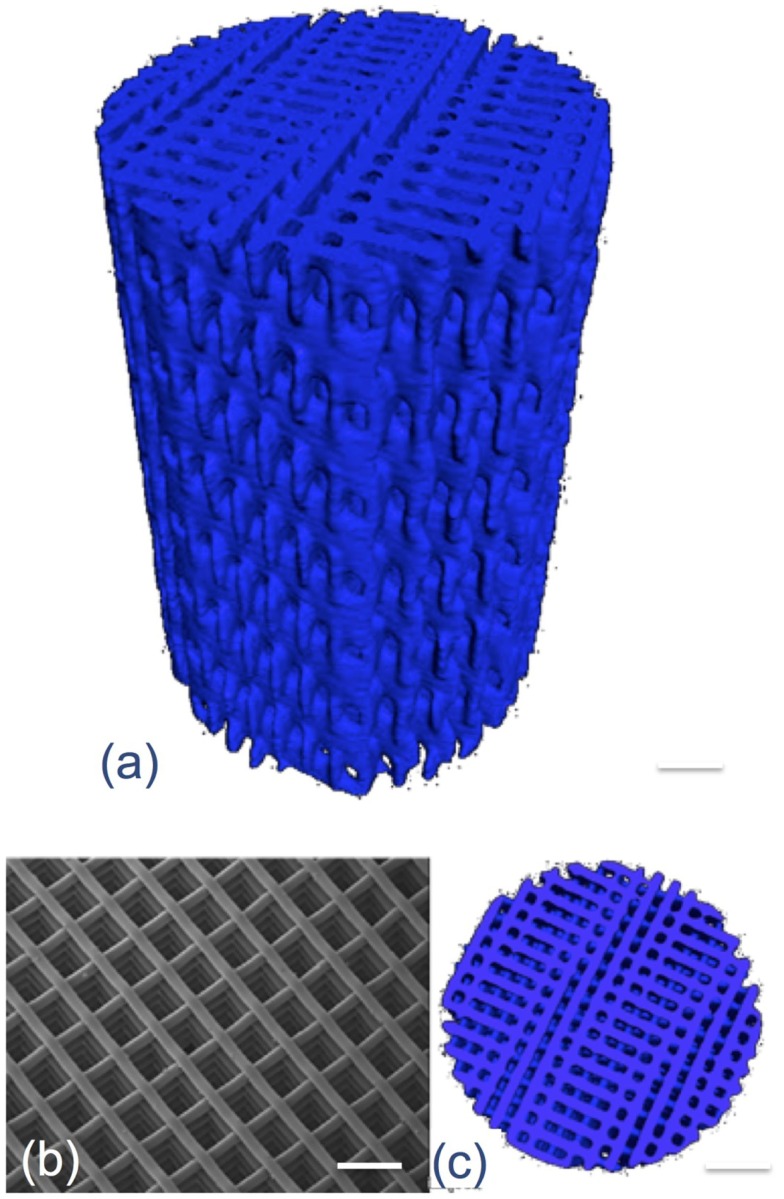
**X-ray microtomography image of 3-D printed bioactive glass scaffolds**. Modified with permission from Jones (2013).

3-D printing has delivered bioactive glass scaffolds with interconnected pores similar in diameter to the porous foam scaffolds developed previously (Figure 4), but with compressive strengths at least an order of magnitude higher, increasing from 2.4 MPa for the foams of 80% porosity (Jones et al., 2006) to >140 MPa for the 3-D printed scaffolds (Fu et al., 2011b). The reason for this is that the layer by layer printing process can deposit thick aligned struts (>50 μm), leaving wide channels in excess of 500 μm, with percentage porosities of 60% (Doiphode et al., 2011; Huang et al., 2011; Kolan et al., 2011).

**Figure 5:**
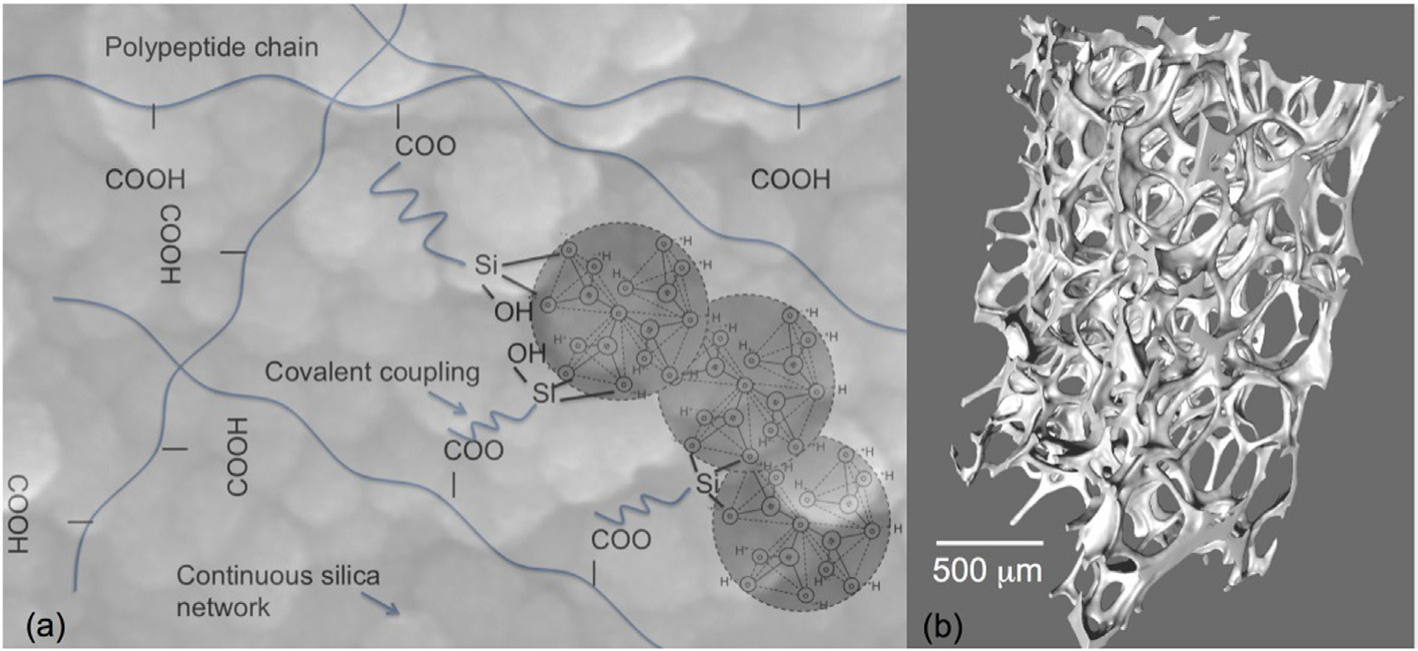
**Sol–gel hybrids: (A) schematic of the concept of inorganic/organic hybrids with bonding between components and (B) X-ray microtomography image of sol–gel foam hybrid scaffolds**. Modified with permission from Jones (2013).

However, bioactive glass scaffolds are still brittle and therefore not suitable for all grafting applications, such as sites that are under cyclic loads. Tougher scaffolds are required that still have all the bioactive properties of Bioglass. One solution is to use composite materials (Rezwan et al., 2006); however, conventional composites of bioactive glass particles can be masked by the polymer matrix and it is difficult to match degradation rates between the polymer and the bioactive glass. An alternative is inorganic/organic hybrids made by the sol–gel process (Sanchez and In, 1992; Novak, 1993; Jones, 2013). As the gelation process occurs at room temperature, polymers can be incorporated into the sol so that the polymer chains are dispersed between the assembling nanoparticles prior to gelation. This provides molecular scale interactions between the components (Figure 5A), which gives the unique potential for control of mechanical properties and degradation rate while providing a homogeneous surface (at the micron scale) for cell attachment (Arcos and Vallet-Regi, 2010; Jones, 2013). In order for congruent degradation to occur, some covalent bonds are needed between the organic and inorganic components (Figure 5A). Examples are silica/natural polymers: e.g., silica/gelatin (Ren et al., 2002; Mahony et al., 2010, 2014), silica/poly(gamma-glutamic acid) (Poologasundarampillai et al., 2010, 2012, 2014; Valliant et al., 2013), silica/chitosan (Shirosaki et al., 2005, 2010; Connell et al., 2014), silica/polyester (Rhee et al., 2002, 2004; Pandis et al., 2015), and silica/PEG (Liu et al., 2012; Russo et al., 2013; Catauro et al., 2015; Li et al., 2015). The foaming method (Figure 5B) can be introduced to the sol–gel hybrid process (Mahony et al., 2010, 2014) or the sol to gel transition can be used to 3-D print the hybrids (Gao et al., 2013). One of the biggest challenges here is to be able to introduce calcium into the silicate network at these low processing temperatures. In sol–gel glass synthesis, using calcium salts, temperatures of 450°C must be surpassed to allow calcium to enter the silicate network, which is too high for organic components. Therefore, alternative methods for calcium incorporation are needed to impart bioactivity (Valliant et al., 2013; Poologasundarampillai et al., 2014).

The frontier that must be crossed is of tuning the mechanical properties and degradation rates of these exciting new materials so that load can be shared with the host tissue and osteogenic cells experience a transfer of load, and mechanical transduction, so that high quality bone regenerates as the scaffold is remodeled by the host tissue.

### Frontier: Use of Inorganic Materials Deliver Ions for Therapeutic Non-Bone Applications

Numerous soft tissue engineering applications have been investigated at an exploratory level but still require development into clinical products (Miguez-Pacheco et al., 2015). Is it possible to obtain regulatory approval for clinical trials of soft tissue applications based upon limited *in vitro* and *in vivo* data and lack of understanding of basic biological mechanisms of soft tissue response to bioactive materials?

## Conclusion

Important frontiers have been crossed where synthetic materials can bond with host bone, preventing fibrous encapsulation and creating a stable implant. Osteogenic cells are stimulated by inorganic bioactive glasses and their dissolution products. Frontiers still to be crossed in orthopedics are advanced bioactive biomaterials that can share load with host bone, transmit the load to the cells, and then degrade as the bone repairs. The concept of controlled delivery of active cations from a bioactive glass works for bone (osteostimulation). Other cations have been shown to stimulate other cells *in vitro*. A frontier to cross is the availability of bespoke bioactive devices for soft tissue therapy. Frontiers are only truly crossed when patients receive the benefits.

## Conflict of Interest Statement

The authors declare that the research was conducted in the absence of any commercial or financial relationships that could be construed as a potential conflict of interest. The Associate Editor Wolfram Höland declared a past coauthorship with the author Julian R. Jones and states that the process, nevertheless, met the standards of a fair and objective review.
